# Sleep Patterns, Alertness, Dietary Intake, Muscle Soreness, Fatigue, and Mental Stress Recorded before, during and after Ramadan Observance

**DOI:** 10.3390/sports7050118

**Published:** 2019-05-17

**Authors:** Omar Boukhris, Khaled Trabelsi, Roy Jesse Shephard, Hsen Hsouna, Raouf Abdessalem, Lassaad Chtourou, Achraf Ammar, Nicola Luigi Bragazzi, Hamdi Chtourou

**Affiliations:** 1 UR15JS01: Education, Motricité, Sport et Santé (EM2S), High Institute of Sport and Physical Education, University of Sfax, Sfax 3000, Tunisia; omarboukhris24@yahoo.com (O.B.); trabelsikhaled@gmail.com (K.T.); hsen.hsouna92@gmail.com (H.H.); raoufabdesalem18@gmail.com (R.A.); 2 Faculty of Kinesiology and Physical Education, University of Toronto, Toronto, Ontario M5S 1A1, Canada; royjshep@shaw.ca; 3 Department of Gastroenterology and Hepatology, Hedi Chaker Hospital, Sfax 3089, Tunisia; dr.chtourou07@yahoo.fr; 4 Institute of Sport Science, Otto-von-Guericke-University Magdeburg, 39104 Magdeburg, Germany; ammar.achraf@ymail.com; 5 Department of Health Sciences (DISSAL), Postgraduate School of Public Health, University of Genoa, 16132 Genoa, Italy; 6 Institut Supérieur du Sport et de l’Education Physique de Sfax, Université de Sfax, Sfax 3000, Tunisia; h_chtourou@yahoo.fr; 7 Activité Physique, Sport et Santé, UR18JS01, Observatoire National du Sport, Tunis 1003, Tunisia

**Keywords:** intermittent fasting, sleep patterns, nutrition, fatigue

## Abstract

Ramadan is one of the pillars of the Islamic creed. Its observance commonly causes chrono-biological changes. The present study examined sleep and alertness during Ramadan observance relative to data collected before and after Ramadan in a sample of young, physically active men. Information was also collected on dietary intake, muscle soreness, fatigue, and mental stress over the three periods. Fourteen physically active men (age: 21.6 ± 3.3 years, height: 1.77 ± 0.06 m, body-mass: 73.1 ± 9.0 kg) completed the Hooper questionnaire and the Pittsburgh Sleep Quality Index (PSQI) and responded to the digit cancellation test (DCT) fifteen days before Ramadan, during the last ten days of Ramadan and 20 days after Ramadan. The PSQI results indicated that sleep duration was significantly longer before Ramadan (p = 0.003) and after Ramadan (p = 0.04) compared to during Ramadan and was longer before Ramadan than after Ramadan (p = 0.04). In addition, the sleep efficiency was lower during Ramadan in comparison to before Ramadan (p = 0.02) and after Ramadan (p = 0.04). The daytime dysfunction score increased during Ramadan in comparison with before Ramadan (p = 0.01) and after Ramadan (p = 0.04), and the sleep quality score was higher during (p = 0.003) and after Ramadan (p = 0.04) as compared to before Ramadan. The sleep disturbance score increased during Ramadan relative to before Ramadan (p = 0.04). However, Ramadan observance had no significant effect on sleep latency. Mental alertness also decreased at the end of Ramadan compared to before (p = 0.003) or after Ramadan (p = 0.01). Dietary intake, muscle soreness, fatigue, and mental stress as estimated by the Hooper questionnaire remained unchanged over the three periods of the investigation (p > 0.05). In conclusion, Ramadan observance had an adverse effect on sleep quantity and on mental alertness, but not on sleep quality. However, dietary intake, muscle soreness, fatigue, and mental stress remained unaffected.

## 1. Introduction

During Ramadan, healthy adult Muslims are required to fast from dawn to sunset over a period of 29 or 30 days. There is abstinence not only from food and drink but also from smoking and other specific behaviors (e.g., sexual activity) [1]. The challenge of maintaining physical training during Ramadan observance is accepted by most Muslim athletes who participate in national and international events (e.g., the National Soccer Championship and Cup and the FIFA World Cup). Training, competition, and eating are all concentrated into the hours of darkness, and this can cause chrono-biological changes that disrupt athlete’s physiology and behavior [2,3], impairing sleep-wake patterns [4,5,6,7,8], increasing fatigue and decreasing physical and mental performance [9,10,11]. Further, the combination of disturbed sleep and changed eating patterns can increase the risk of injury [12].

Many sport disciplines require a high level of attention to manage and resolve problem situations that arise during play [13]. The digit cancellation test (DCT) provides information about executive functioning and the ability to focus attention [14]. Chamari et al. [4] and Zarrouk et al. [15] found no significant changes in the alertness of athletes during Ramadan observance, but other investigators have noted a deterioration in various aspects of psychomotor performance, subjective alertness and memory [8,15,16,17,18,19]. The Hooper questionnaire is a useful instrument that may help to detect individual signs of pre-fatigue and mental stress [20,21], potentially exacerbated by participating in training and/or competitions in a fasted and dehydrated state. Use of the Hooper questionnaire and careful dietary assessment could potentially contribute to the health and performance of an athlete during Ramadan observance.

A deterioration of mood state and loss of sleep could also contribute to a decrease in performance during Ramadan [22,23,24]. To date, studies evaluating the impact of Ramadan observance on the stress level of athletes are lacking, but such information would help coaches and trainers to devise tactics to combat stress and maintain a normal level of vigilance.

Thus, the present study examined the impact of Ramadan observance on sleep patterns, mental alertness, dietary intake, muscle soreness, fatigue, and mental stress, comparing data obtained before, during and after Ramadan observance. The hypothesis was that, in a sample of young subjects, the alterations of sleep quantity, quality and dietary changes occurring during Ramadan would adversely affect alertness, muscle soreness, fatigue, and mental stress.

## 2. Materials and Methods 

### 2.1. Participants

The software G∗power [25] was used a priori to calculate the necessary minimum sample size, based on procedures suggested by Beck [26]. Values for α were set at 0.05 and for power at 0.95. Based on an earlier study of Herrera et al. [27] and discussions between the authors, effect sizes were estimated as 0.46. In total, to reach the desired power, data from at least eleven participants were deemed to be sufficient to minimize the risk of incurring a type 2 statistical error.

This sample size was computed specifically concerning the sleep test and may not adequately capture statistically significant differences regarding other variables studied, such as dietary intake.

Fourteen physically active men (age: 21.6 ± 3.3 years, height: 1.77 ± 0.06 m, body-mass: 73.1 ± 9.0 kg) volunteered for this study. They were not members of formal sports teams, but practiced physical activity regularly (e.g., jogging) for at least ≈1 h per day, three days per week. The usual time of exercising was 17h00, and this was not modified in volume (i.e., ~8 km of jogging in 1 h) or in timing during Ramadan. All participants were non-smokers, did not consume alcohol, had no pathological sleep disorders and did not work during the entire period of investigation. 

All initially signed an informed consent form. The protocol respected the Helsinki convention, and was approved by the Research Ethics Committee “CPP SUD N°0098.” The study was carried out in Tunisia in 2016, with Ramadan beginning on 6 June and concluding on 5 July. The average environmental conditions were a temperature of around 28 °C and relative humidity of 50% before Ramadan, 32 °C and 49% during the last ten days of Ramadan, and 31 °C and 47% during the 20 days after Ramadan.

### 2.2. Experimental Design

Participants visited the laboratory on three separate occasions: before Ramadan, during the end of Ramadan and after Ramadan. In the afternoon of each occasion (between 16.30 and 18.00), participants completed a validated Arabic version of the Pittsburgh Sleep Quality Index [28], the digit cancellation test of mental alertness, and the Hooper stress questionnaire. Dietary intake was also assessed at each of the three visits.

#### 2.2.1. The Pittsburgh Sleep Quality Index 

The validated Arabic version of the Pittsburgh Sleep Quality Index [28] assessed subjective sleep quality over the previous month [29]. It comprised 19 questions, covering seven components of sleep: duration, quality, latency, efficiency, disturbances, daytime dysfunction, and the use of sleeping medications. The total score ranges from 0 to 21, where “0” designates no troubles and “21” indicates severe problems in all areas of sleep.

#### 2.2.2. The Digital Cancellation Test

The digit cancellation test provides a highly practical and user-friendly assessment of various aspects of prefrontal cortical functioning, including information processing speed, the ability to focus attention and executive functioning [14]. Participants performed the test by crossing out target numbers (i.e., numbers composed by three digits) on a sheet of randomly arranged numbers. The score was given by the sum of the numbers correctly canceled in one minute.

#### 2.2.3. Dietary Intake

Participants completed a 10-day diary noting all food and beverages consumed during this period. Diaries were analyzed by an experienced nutritionist from the CHU Hedi Chaker, using an appropriate computer program (Nutrisoft-Bilnut, version 2.01) and the food-composition tables of the Tunisian National Institute of Statistics (1978).

#### 2.2.4. The Hooper Questionnaire

The Hooper questionnaire detects signs of pre-fatigue and stress [20,21]. Participants provided information about their subjective assessment (i.e., feelings) of sleep quality the previous night, as well as ratings of fatigue, stress, and muscle soreness. Each response was rated on a seven-point Likert scale, with responses ranging from “very, very good” to “very, very bad” for sleep, and from “very, very low” to “very, very high” for fatigue, stress, and muscle soreness. The Hooper Index reflects the sum of the four ratings.

### 2.3. Statistical Analyses

Data were analyzed using Statistica software (StatSoft, Paris, France, version 10). The measured parameters were expressed as means ± SDs. The normality of data distribution was first verified by the Shapiro–Wilk test. Values for the total score of the Pittsburgh sleep questionnaire, the digital cancellation test, the stress registered by the Hooper questionnaire, and dietary intake (i.e., the intake of saturated fats, monounsaturated fats, polyunsaturated fats, carbohydrates, proteins, total energy, magnesium, and vitamin B1) were all normally distributed, allowing use of parametric tests, including a one-way ANOVA (3 time periods). When appropriate, post hoc comparisons were made using the Bonferroni test. Effect sizes were calculated as partial eta-squared *ɳ*_p_^2^ with values of 0.01, 0.06, and 0.13 considered as small, moderate, and large, respectively.

Scores for sleep duration, quality, latency, efficiency, sleep disturbances, and daytime dysfunction, as well as data for fatigue, sleep and muscle soreness as registered by the Hooper questionnaire and the dietary intake of saccharose, cholesterol, potassium, sodium, iron, zinc, vitamin E, calcium, phosphorus, folate, vitamin C, and fibers were not normally distributed. Data for these items were thus evaluated using a Friedman non-parametric analysis of variance (ANOVA), with estimations of effect size based on Kendall’s coefficient of concordance. When significant, pair-wise comparisons were conducted using a Wilcoxon test.

In addition, Bland and Altman correlations were used to examine relationships between the measured variables. As suggested by Bland and Altman [30], repeated measures correlation is a statistical technique for determining the common within individual association for paired measures assessed on two or more occasions for multiple individuals. Significance was accepted for all analyses at the level of p < 0.05.

## 3. Results

### 3.1. The Pittsburgh Sleep Quality Index

Data of the Pittsburgh Sleep Quality Index are reported in Table 1, before, during and after Ramadan.

The Friedman test revealed significant main effects of time periods on sleep efficiency (test = 9.30, p = 0.009, Kendall’s W = 0.33), sleep duration (test = 13.60, p = 0.001, Kendall’s W = 0.48), sleep quality (test = 14.82, p = 0.0006, Kendall’s W = 0.52), sleep disturbances (test = 7.60, p = 0.02, Kendall’s W = 0.27), and daytime dysfunction (test = 7.91, p = 0.01, Kendall’s W = 0.28). Pair-wise comparisons indicated that sleep efficiency was lower during Ramadan in comparison to before Ramadan (p = 0.02) and after Ramadan (p = 0.04). Sleep duration was significantly higher before Ramadan (p = 0.003) and after Ramadan (p = 0.04) compared to during Ramadan and was higher before Ramadan than after Ramadan (p = 0.04). The sleep quality score was higher during (p = 0.003) and after Ramadan (p = 0.04) as compared to before Ramadan. The sleep disturbance score also increased significantly during Ramadan relative to before Ramadan (p = 0.04). The daytime dysfunction score was found to increase during Ramadan in comparison with before Ramadan (p = 0.01) and after Ramadan (p = 0.04). However, Ramadan observance had no significant effect on sleep latency (Friedman test = 1.65, p = 0.43, Kendall’s W = 0.05) (Table 1).

There was a significant main effect of time on the total score for the Pittsburgh questionnaire (F = 21.94, p < 0.0005, *ƞ_p_^2^* = 0.62, Table 1). Post hoc analyses showed a significantly higher total score during Ramadan in comparison with before Ramadan (p < 0.0005) or after Ramadan (p = 0.004). The total score for this questionnaire was also somewhat higher after Ramadan compared to before Ramadan (p = 0.01) (Table 1).

### 3.2. The Digit Cancellation Test

Ramadan observance had a significant main effect on the digit cancellation test (F = 10.77, p < 0.0005, *ƞ_p_^2^* = 0.45). Post hoc analysis showed that scores were lower at the end of Ramadan compared to either before Ramadan (p < 0.0005) or after Ramadan (p = 0.01) (Table 2).

### 3.3. The Hooper Questionnaire

There was no significant main effect of Ramadan observance on stress (F = 1.16, p = 0.32, *ƞ_p_^2^* = 0.08), fatigue (test = 4.55, p = 0.10, Kendall’s W = 0.16), sleep (test = 4.68, p = 0.09, Kendall’s W = 0.16) or muscle soreness (test = 2.90, p = 0.23, Kendall’s W = 0.10) as assessed by the Hooper questionnaire. The overall Hooper index showed a significant main effect of time (F = 3.41, p = 0.04, *ƞ_p_^2^* = 0.20), but post hoc analysis established that the Hooper index values recorded during the three periods did not differ significantly from each other (Table 2).

### 3.4. Dietary Intake

There was no significant main effect of time on any of the dietary variables, including total energy intake (F = 1.74, p = 0.19, *ƞ_p_^2^* = 0.11), carbohydrate intake (F = 2.29, p = 0.12, *ƞ_p_^2^* = 0.14), protein intake (F = 0.27, p = 0.76, *ƞ_p_^2^* = 0.02), fat intake (F = 2.43, p = 0.10, *ƞ_p_^2^* = 0.15), saturated fat (F = 3.25, p = 0.054, *ƞ_p_^2^* = 0.19), monounsaturated fat (F = 0.04, p = 0.95, *ƞ_p_^2^* = 0.003), polyunsaturated fat (test = 2.87, p = 0.23, Kendall’s W = 0.10), saccharose (test = 4.53, p = 0.10, Kendall’s W = 0.16), cholesterol (test = 0.57, p = 0.75, Kendall’s W = 0.02), potassium (test = 3.0, p = 0.22, Kendall’s W = 0.10), sodium (test = 5.57, p = 0.06, Kendall’s W = 0.19), magnesium (F = 1.72, p = 0.19, *ƞ_p_^2^* = 0.11), iron (test = 3.42, p = 0.18, Kendall’s W = 0.12), zinc (test = 0.15, p = 0.92, Kendall’s W = 0.005), vitamin E (test = 1.14, p = 0.56, Kendall’s W = 0.04), vitamin B1 (F = 1.27, p = 0.29, *ƞ_p_^2^* = 0.08), calcium (test = 1.85, p = 0.39, Kendall’s W = 0.06), phosphorus (test = 1.12, p = 0.56, Kendall’s W = 0.04), folate (test = 1.41, p = 0.49, Kendall’s W = 0.05), vitamin C (test = 0.98, p = 0.61, Kendall’s W = 0.03) or fibers (test = 4.27, p = 0.11, Kendall’s W = 0.15) (Table 3).

### 3.5. Bland and Altman Correlations

Bland and Altman correlations revealed that many characteristics of sleep were correlated with digital cancellation scores and with stress, fatigue, sleep and the overall Hooper questionnaire score. Digital cancellation was significantly correlated with sleep duration (r = 0.43, p = 0.01), sleep efficiency (r = 0.45, p = 0.01), daytime dysfunction (r = 0.43, p = 0.01), sleep disturbances (r = 0.48, p = 0.009), and the total Pittsburgh questionnaire score (r = 0.53, p = 0.003).

Stress was significantly correlated with sleep duration (r = 0.61, p < 0.0005), sleep efficiency (r = −0.38, p = 0.04), sleep quality (r = 0.54, p = 0.002), sleep disturbances (r = −0.62, p < 0.0005), daytime dysfunction (r = 0.40, p = 0.03), and the total Pittsburgh questionnaire score (r = −0.69, p < 0.0005).

Fatigue was significantly correlated with sleep duration (r = −0.48, p = 0.008), sleep efficiency (r = −0.49, p = 0.006), sleep quality (r = 0.42, p = 0.02), sleep disturbances (r = −0.56, p = 0.001), daytime dysfunction (r = 0.57, p = 0.001), and the total Pittsburgh questionnaire score (r = 0.71, p < 0.0005).

The Hooper sleep assessment was significantly correlated with sleep duration (r = −0.61, p < 0.0005), sleep efficiency (r = −0.38, p = 0.04), sleep quality (r = 0.54, p = 0.002), sleep disturbances (r = 0.62, p < 0.0005), daytime dysfunction (r = 0.40, p = 0.03), and total Pittsburgh score (r = 0.69, p < 0.0005).

Muscle soreness was significantly correlated with sleep quality (r = 0.37, p = 0.04), sleep disturbances (r = 0.48, p = 0.007) and total Pittsburgh score (r = 0.43, p = 0.01). 

The Hooper index was significantly correlated with sleep duration (r = −0.47, p = 0.009), sleep efficiency (r = −0.42, p = 0.02), sleep quality (r = 0.44, p = 0.01), sleep disturbances (r = 0.66, p < 0.0005), daytime dysfunction (r = 0.44, p = 0.01), and the total Pittsburgh score (r = 0.66, p < 0.0005).

## 4. Discussion

The major finding from the present study was that Ramadan observance had negative effects upon sleep quantity and mental alertness in physically active, young men. However, the levels of stress, fatigue, sleep and muscle soreness indicated by the Hooper questionnaire remained unchanged, as did the estimated dietary intake. Sleep quality as indicated by the Pittsburgh sleep quality questionnaire was higher during and after Ramadan compared to before Ramadan.

Several previous investigations have reported alterations of alertness, memory, reaction time and psychomotor performance during Ramadan observance [8,31,32,33]. However, the present findings disagree with earlier data in showing that cognitive function was unaffected by Ramadan observance [34,35]. Many factors can affect cognitive function negatively, including sleep loss, shifts of circadian rhythm, and impaired motivation, as well as the dehydration and decreased blood glucose levels inevitable in the late afternoons of Ramadan [1]. 

Ramadan observance could cause cumulative deficits of food and water intake, but in the present study, intakes did not change significantly, in agreement with Aziz et al. [36] and the findings of a recent systematic review [23]. Night-time sleep is often delayed, and its duration tends to be reduced because of the large amounts of food that are consumed late at night [37]. The present study observed that digit cancellation scores were significantly correlated with several measures, including sleep duration and efficiency, daytime dysfunction, and the total Pittsburgh score. These correlations support the hypothesis that the reduction of alertness during Ramadan is related to disruptions in the quantity of sleep. Sleep deprivation leads to a slowing of reaction times and attentional deficits, particularly in the late afternoon [38,39,40], and this could well explain the decrease of alertness that we observed. Others have shown that sleeping less than the recommended eight hours generates deficits of cognitive performance [41]. In the present study, the average estimated sleep duration was only about 6.1 h, well below the recommended average [41]. However, the impact of Ramadan upon sleep has varied widely from one study to another. Bouhlel et al. [34] and Chamari et al. [4] found that sleep duration was well maintained during Ramadan observance, and this could explain why they saw no significant changes in cognitive performance.

If a sleep deficit accumulates over the course of Ramadan, it can cause mood swings, with feelings of lethargy, and overall fatigue [42]. In the present study, Hooper estimates of stress, sleep, fatigue and muscle soreness were not adversely affected by Ramadan observance. In contrast, previous studies have shown increased subjective ratings of fatigue on the Profile of Mood State scale [43,44] and the Hooper questionnaire [45]. The present results showed that fatigue (as estimated by Hooper index) and the overall Hooper index significantly correlated with the sleep parameters of the Pittsburgh questionnaire. In agreement with these findings, others have shown that sports performance and mood states bear a direct relationship to sleep quality [46,47,48,49,50,51,52]. Adequate sleep seems essential to optimal athletic performance, with its positive effects on homeostatic neuroendocrine and immune mechanisms and physical and emotional recovery [52,53,54].

The results of the present study did not show an increase in stress, as estimated by the Hooper questionnaire. A recent study that was carried out on sedentary subjects suggested that for some people, religious practices such as fasting in the holy month of Ramadan can even reduce stress, anxiety, and depression [55]. Measurements of tension, depression, and anger using the profile of Mood States have also remained unchanged during Ramadan observance [43,44].

The digit cancellation scores had returned to their pre-Ramadan values by 20 days after Ramadan, in tandem with the normalization of scores for sleep efficiency, sleep disturbances and daytime dysfunction.

The present findings apply only to young men who are engaging in a moderate weekly volume of aerobic activity. Very different findings might be seen in elite athletes, facing a much higher daily level of physical activity, coupled with the stresses of international competition, regulation of their lifestyles by coaches and trainers, and often communal living in athletes villages, possibly with difficulty in obtaining meals at times compatible with the observance of Ramadan. Thus, future studies should re-examine in high-level athletes the effects upon sleep and alertness that we have observed. One other obvious limitation to the present study is the lack of a control group who did not fast during the month of Ramadan. In most Muslim countries, the non-fasters tend not to proclaim themselves as non-fasters, and for this reason, are difficult to recruit. Future studies would also be enhanced by objective measures of sleep parameters, such as the use of actigraphs or polysomnography.

Moreover, as previously specified, the sample size was a priori computed specifically concerning the sleep test and may not adequately capture statistically significant differences regarding other variables studied, such as dietary intake. As such, further studies with larger sample sizes are warranted. 

Finally, the present findings specifically refer to a specific denomination population (namely, young, physically active men) and, as such, caution should be paid when interpreting and generalizing the current results. Moreover, other denomination populations should be object of future ad hoc investigations. 

## 5. Conclusions

Young men engaging in a regular moderate volume of daily aerobic activity show disturbances of many aspects of sleep quantity, with an accompanying deterioration of alertness as evaluated by the digital cancellation test, but not of sleep quality, which was found to be higher during and after the Ramadan compared to before Ramadan, as indicated by the Pittsburgh sleep questionnaire. However, stress, fatigue, and muscle soreness, as assessed by the Hooper questionnaire were not modified by Ramadan observance, and the total daily intake of energy, carbohydrate, fat and protein intake remained unchanged. All sleep and alertness parameters had returned baseline values by 20 days after the conclusion of Ramadan.

## Figures and Tables

**Table 1:** Subjective sleep quality, as recorded before, during and after Ramadan.

	Before Ramadan	During Ramadan	After Ramadan
**Sleep latency (min)**	16.8 ± 6.4	17.6 ± 6.4	17.6 ± 6.1
**Sleep efficiency (%)**	98.4 ± 4.1	93.3 ± 8.2*#	96.4 ± 6.0
**Sleep duration (h)**	7.9 ± 1.6	6.1 ± 1.5*#	6.9 ± 1.4*
**Sleep quality**	0.9 ± 0.9	2.0 ± 0.7*	1.6 ± 0.9*
**Sleep disturbances**	0.6 ± 0.5	0.9 ± 0.5*	0.7 ± 0.5
**Daytime dysfunction**	0.4 ± 0.6	0.8 ± 0.8*#	0.3 ± 0.5
**Total score of PSQI**	3.3 ± 2.3	6.7 ± 2.6*#	4.9 ± 2.0*

* Significant differences from before Ramadan. # Significant differences from after Ramadan.

**Table 2:** Digit cancellation test and Hooper parameters (i.e., fatigue, stress, sleep, muscle soreness, and overall Hooper index) measured before Ramadan, during the last ten days of Ramadan and twenty days after Ramadan.

	Before Ramadan	End of Ramadan	After Ramadan
**Fatigue**	3.3 ± 1.5	4.3 ± 1.8*	3.6 ± 1.4
**Stress**	2.9 ± 1.4	3.5 ± 2.0	2.8 ± 1.6
**Sleep**	3.1 ± 1.5	4.6 ± 1.7*	3.9 ± 1.2
**Muscle soreness**	3.0 ± 1.7	3.8 ± 1.4#	2.9 ± 0.9
**Overall Hooper index**	12.3 ± 5.4	16.1 ± 6.2	13.2 ± 2.9
**DCT**	66 ± 10	60 ± 11*#	64 ± 10

* Significant differences in comparison with before Ramadan. # Significant differences in comparison with after Ramadan.

**Table 3:** Estimated daily dietary intake (mean ± SD) during fifteen days before Ramadan, the last ten days of Ramadan and twenty days after Ramadan.

	Before Ramadan	End of Ramadan	After Ramadan
**Total energy intake (MJ/day)**	11.2 ± 2.0	10.7 ± 1.2	10.4 ± 2.3
**Carbohydrate (g/kg/day)**	4.7 ± 1.0	4.2 ± 1.1	4.5 ± 1.2
**Protein intake (g/kg/day)**	1.1 ± 0.4	1.1 ±0.3	1.1 ± 0.3
**Total fat intake (g/kg/day)**	1.6 ± 0.4	1.7 ± 0.3	1.4 ± 0.4
**Saturated fat (%)**	27.9 ± 4.1	26.7 ± 3.1	30.2 ± 5.4
**Monounsaturated fat (%)**	50.7 ± 6.8	51.4 ± 6.9	50.8 ± 6.9
**Polyunsaturated fat (%)**	19.1 ± 6.7	21.7 ± 8.2	18.1 ± 6.2
**Saccharose (g)**	38.9 ± 17.9	37.9 ± 40.5	36.4 ± 21.8
**Cholesterol (g)**	433.9 ± 163.7	463.6 ± 191.6	407.9 ± 172.0
**Potassium (mg)**	2375.8 ± 562.8	2216.8 ± 750.4	2531.4 ± 567.4
**Sodium (mg)**	4464.5 ± 2075.8	5199.6 ± 2311.3	3685.4 ± 2123.1
**Magnesium (mg)**	226.4 ± 62.2	201.8 ± 47.0	241.1 ± 61.9
**Iron (mg)**	12.0 ± 3.2	13.7 ± 4.8	10.9 ± 2.2
**Zinc (mg)**	10.3 ± 3.2	9.5 ± 3.2	9.5 ± 2.3
**Vitamin E (mg)**	8.1 ± 2.9	8.0 ± 2.1	7.1 ± 3.1
**Vitamin B1 (mg)**	0.5 ± 0.1	0.5 ± 0.1	0.5 ± 0.1
**Calcium (mg)**	679.1 ± 366.6	684.3 ± 227.9	577.4 ± 194.7
**Phosphorus (mg)**	1309.2 ± 350.6	1174.4 ± 235.9	1168.2 ± 258.0
**Folate (mg)**	208.4 ± 58.7	197.9 ± 97.8	207.1 ± 71.8
**Vitamin C (mg)**	50.1 ± 28.2	34.6 ± 27.5	48.1 ± 30.7
**Fibers (mg)**	16.7 ± 5.1	13.4 ± 5.4	16.2 ± 5.7
